# Non-additive genome-wide association scan reveals a new gene associated with habitual coffee consumption

**DOI:** 10.1038/srep31590

**Published:** 2016-08-25

**Authors:** Nicola Pirastu, Maarten Kooyman, Antonietta Robino, Ashley van der Spek, Luciano Navarini, Najaf Amin, Lennart C. Karssen, Cornelia M Van Duijn, Paolo Gasparini

**Affiliations:** 1 Institute for Maternal and Child Health IRCCS Burlo Garofolo, Trieste Italy; 2 University of Trieste, Trieste, Italy; 3 Usher Institute of Population Health and Informatics, The University of Edinburgh, Edinburgh, UK; 4 Genetic Epidemiology Unit, Department of Epidemiology, Erasmus Medical Center, Rotterdam, the Netherlands; 5 illycaffe’s.p.a, Trieste, Italy; 6 PolyOmica, Groningen, The Netherlands; 7 Centre for Medical Systems Biology, Leiden University Medical Center, Leiden, The Netherlands

## Abstract

Coffee is one of the most consumed beverages world-wide and one of the primary sources of caffeine intake. Given its important health and economic impact, the underlying genetics of its consumption has been widely studied. Despite these efforts, much has still to be uncovered. In particular, the use of non-additive genetic models may uncover new information about the genetic variants driving coffee consumption. We have conducted a genome-wide association study in two Italian populations using additive, recessive and dominant models for analysis. This has uncovered a significant association in the PDSS2 gene under the recessive model that has been replicated in an independent cohort from the Netherlands (ERF). The identified gene has been shown to negatively regulate the expression of the caffeine metabolism genes and can thus be linked to coffee consumption. Further bioinformatics analysis of eQTL and histone marks from Roadmap data has evidenced a possible role of the identified SNPs in regulating PDSS2 gene expression through enhancers present in its intron. Our results highlight a novel gene which regulates coffee consumption by regulating the expression of the genes linked to caffeine metabolism. Further studies will be needed to clarify the biological mechanism which links PDSS2 and coffee consumption.

Coffee is one of the most widely drunk beverages, being second only to water and tea^1^. Given that it contains many different physiologically active compounds such as caffeine, polyphenols (e.g., chlorogenic acids), niacin, N-methylpyridinium ion and others^2^, many studies have investigated its impact on health, finding associations with common disorders. For example, coffee consumption has been linked to protective effects on various common pathologies such as cardiovascular diseases^3^, hypertension^4^,^5^, Alzheimer’s and Parkinson’s diseases^6^,^7^, type 2 diabetes^8^,^9^,^10^, some types of cancer^11^,^12^ and hearing functions^13^, while it may predispose to others such as sleep disturbances^14^,^15^. The earliest studies on the genetic bases of coffee consumption can be dated back as far as the 1960’s with the first description of its heritability in Italy^16^, which has been estimated to range between 0.36 and 0.58 in twin studies. Different independent genome-wide association studies (GWAS) carried out mostly in Northern European populations have highlighted associations between coffee or caffeine consumption and several genes: *CYP1A1-CYP1A2*^17^,^18^, *AHR*^17^ *NRCAM* and *ULK3*^18^ while moderate association has been seen with the adenosine receptor A2, which is actually one of the effector proteins of caffeine^17^. More recently, a very large study comprising more than 120 thousand people has confirmed some of these (*CYP1A1-CYP1A2* and *AHR*) while identifying 6 novel ones^19^. Despite these successes much of the heritability of coffee consumption still remains unexplained, part of which could be explained by the fact that the additive genetic model is almost always used in association studies, whereas, in fact, genes can exhibit recessive or dominant effects. It is in fact very well known that miss-specifying the genetic model will result in a considerable loss of power, especially under certain conditions^20^. For this reason we have decided do conduct a genome-wide association study looking for non-additive effects on two isolated populations of Italy in order to verify whether genes are affecting coffee consumption under these genetic models.

## Materials and Methods

### Study Populations and study design

For the discovery phase we used the samples from two isolated Italian populations participating to the INGI consortium. In particular, our study used 370 individuals from INGI-CARL, a population coming from Carlantino, a small village located in Puglia (Southern Italy), and 843 defined as INGI-FVG, making reference to 6 villages situated in the Friuli Venezia Region in North-Eastern Italy for a total of 1207 samples. For replication we used the samples coming from the Erasmus Rucphen Family (ERF) study, a cross-sectional cohort including 3,000 living descendants of 22 couples who had at least 6 children baptized in the community church around 1850–1900; 1731 samples were used from this study.

### Phenotype and sample selection

Coffee consumption was assessed in the INGI cohorts through field interviews while it was self-reported in the ERF cohort. In all cohorts coffee consumption was measured as number of coffee cups per day. Given that the distribution of the trait was extremely skewed we transformed this to log_10_(*N*_coffee cups_ + 1). In order to remove outliers, we excluded people who drank more than 9 cups of coffee per day in the Italian cohorts and more than 20 in ERF. This difference was due to the very different coffee consumption distribution observed in the Italian vs. the Dutch populations. Finally, all people under hypertensive medication were excluded from the analysis since those are usually advised to reduce or avoid coffee consumption.

### Genotyping and Imputation

Genotyping was carried out as previously described^21^,^22^. Briefly, INGI-CARL, and INGI-FVG have been genotyped with the Illumina 370k high density SNP array. Genotype imputation on the INGI cohorts was conducted after standard QC using SHAPEIT2^23^ for the phasing step and IMPUTE2^24^ for the imputation using the1000 Genomes phase I v3 reference set^25^. ERF has been genotyped with different genotyping platforms: Illumina 318 k, 350 k, 610 k and Affymetrix 200 k. Genotypes were pooled together after QC, phased and imputed to the 1000 Genomes dataset phase I v3^26^ using MaCH and minimac^27^. After imputation SNPs with MAF <0.01 or Info score <0.4 we excluded from the statistical analyses for each of the populations but ERF, for which R^2^ < 0.3 was used instead.

### Association analysis

Association analysis was conducted using mixed model linear regression where the standardized food liking was used as the dependent variable and the SNP dosages as the independent variable. Sex and age were used as covariates. The kinship matrix based on all available genotyped SNPs was used as the random effect. For ERF the kinship matrix was estimated on 14.4 k SNPs common to all different genotyping platforms used. Association analysis was conducted using the FASTA method^28^ as implemented in the GenABEL 1.7–2^29^ R package in order to eliminate the effect of familial relatedness from the trait. MixABEL^30^ was used for the actual association of the imputed SNPs. SNPs that did not pass quality control for more than one population were discarded. It is common practice in GWAS to run association presuming the genetic variants have an additive effect on the phenotype, however this is not necessarily true. Therefore, we decided to also use non-additive genetic models, in particular the dominant and recessive models. For the discovery step, association analysis was conducted separately for each INGI cohort and results were pooled using the inverse-variance weighting method. Given that no meta-analysis software supports non-additive genetic models we developed custom R scripts. After meta-analysis in the INGI cohorts, genomic control was used to eliminate any residual stratification and all significant SNPs where taken forward to the replication step in the ERF cohort. The significance threshold was estimated considering the number of equivalent tests performed when running multiple genetic models, which has been estimated as being 2.2^31^, and was thus set at 2.27 × 10^−8^. In order to avoid false positive results due to rare categories, in each GWAS we eliminated those SNPs in which the coded category was either <0.01 or >0.99. This resulted in a different number of SNPs depending on the model used. Supplementary Table S2 summarizes the number of SNPs used for each cohort under each model.

### Comparison of the association pattern of coffee consumption with eQTL results

In order to verify if the observed SNP association pattern could be traced back to an expression quantitative locus (eQTL), we have decided to compare it with that coming from the cis-eQTL of *PDSS2* gene available from 14 different tissues from the GTEx database^32^. We have downloaded the full dataset of results and extracted all SNPs tested against the *PDSS2* gene which fell inside the two recombination hotspots delimiting our association signal (chr6:107400000-107900000). If the two association signals can be traced back to each other we would expect that the strengths of the association should resemble each other and the relationships between the direction of their effect should be consistent across all SNPs examined and in particular in those showing the lowest *p*-values. In order to give more weight to the SNPs with the lowest *p*-values and to keep the directionality of the effect, we assigned to each SNP a score equal to −log_10_(*p*-value) × 1 if the direction of the effect was positive and log_10_(*p*-value) otherwise. Given that the two association patterns do not have a normal distribution we used the Kendal 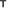 and correlation test to verify if the two patterns resembled each other and if the resulting correlation was significant.

### Functional annotation of the discovered SNPs

In order to understand the possible functional role of the 5 replicated SNPs we annotated them using Haploreg v4.1^33^. Briefly, Haploreg allows one to annotate a list of SNPs by reporting the associated chromatin stated on a 15-state model from the using the Roadmap epigenomics project^34^ and Encode project^35^. Moreover it checks if the SNPs have been previously associated to any eQTL, QTL or metabolite levels according to various databases. Finally, it allows one to set an LD threshold based on which all SNPs with r^2^ > threshold will be included in the annotation. For this study we limited the analysis to those SNPs that have r^2^ > 0.8 with our 5 SNPs. The results from this analysis can be found in Supplementary Table S4.

### Ethical statement

All studies adhered to the tenets of the Declaration of Helsinki. The ERF study was approved by the Medical Ethics Committee of the Erasmus Medical Center in Rotterdam. Informed consent was obtained after explanation of the nature and possible consequences of the study.

All subjects in the INGI-CARL and INGI-FVG studies provided written informed consent before participation. Approval for the research protocol was obtained from the ethical committee of IRCCS-Burlo Garofolo Hospital.

## Results

Supplementary Table S1 summarizes the population characteristics for each of the studies used. The Italian populations had comparable composition and distribution of coffee consumption. In ERF we detected a much higher coffee consumption with a mean more than double (5.6 cups per day vs 1.9 in INGI-CARL and 2.3 in INGI-FVG) that of the other populations and a much higher standard deviation (3.6 vs 1.6 in both INGI populations).

**Figure 1:**
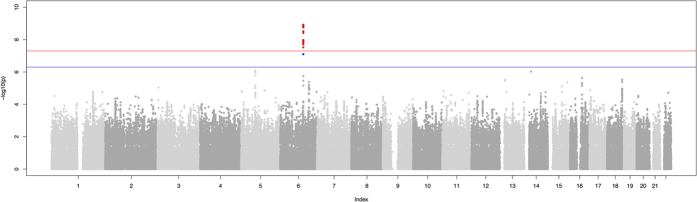
Manhattan plot of the genome-wide association analysis under the recessive model.

**Figure 2:**
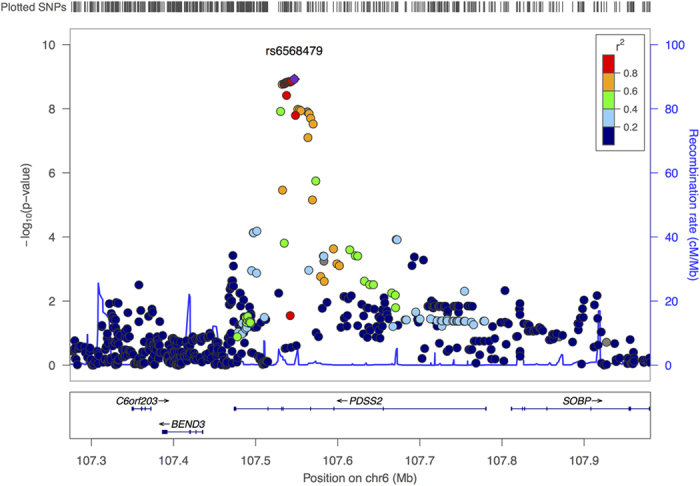
Regional plot of the results from the association analysis around the most significant SNPs.

None of the discovery step genome-wide associations revealed evidence of residual stratification either in each separate cohort or after meta-analysis (genomic control λ between 0.99 and 1.02, see Table S2 for details). Meta-analysis between the results from the INGI cohorts revealed 21 SNPs under the recessive model to be genome-wide significant, with the top hit rs6568479 showing a *p*-value = 8.9 × 10^−10^ and an effect of 0.086 corresponding to a difference of 1.2 cups of coffee per day for the people homozygous for the G allele. No association were found using the dominant genetic model. Table 1 summarizes the results of the association analysis and replication for the 21 significant SNPs. Effect sizes in the two populations were extremely similar (CARL 0.0864, FVG 0.0861). Some of the SNPs showed significant results also under the additive model although *p*-values were higher. For this reason, we consider the recessive model to be the true genetic model. Figure 1 shows the Manhattan plot from the recessive model meta-analysis. As can be seen in Fig. 2 all SNPs fell in the same locus in chromosome 6 all inside the *PDSS2* gene which codes for the coenzyme Q10 (Fig. 2 shows the regional plot for the same SNPs). Replication of the results from the 21 significant SNPs in the ERF cohort revealed that 5 out of 21 SNP had a *p* < 0.05 with a concordant although attenuated effect. All 5 SNPs were significant after pooling the results from the three cohorts with a *p*-value lower than the one observed in the discovery step. None of the identified SNPs are coding, however we sought to verify if any of these could be associated with an existing e-QTL of *PDSS2*. For this reason we downloaded the full results from the Gtex Database^32^ for all cis-eQTL SNPs tested against *PDSS2* falling inside the two recombination hot-spots delimiting the association signal. Comparing the patterns of association for all the SNPs (see Materials and Methods for details) between the two recombination hot spots that show significant correlation with all the association patterns for the tested tissues shows correlation values varying between −0.27 for Adipose Subcutaneous tissue and −0.61 for Esophagus Mucosa tissue. In all cases correlation was negative indicating that people with a higher consumption of coffee have a lower expression of *PDSS2*. Supplementary Table S3 reports the Kendall’s 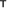 values for each tissue. The annotation with Haploreg showed 7 further SNPs in strong LD with the 5 replicated ones which were not in included in the association analysis. Although none of the 12 SNPs resulted to be coding, 6 out of 12 are located inside enhancers regions active in numerous tissues (Table S4). Finally consistently with the analysis on the Gtex Database all SNPs showed significant association with tissue specific expression of PDSS2.

## Discussion

In this study we have highlighted a novel association between coffee consumption and the *PDSS2* gene. Moreover we have shown that the association pattern correlates very strongly with the expression QTLs in different tissues. Finally the bioinformatics analysis shows that several of the identified SNPs are either in or in LD with enhancers sequences active in various tissues. All these results would suggest that the SNPs would act on coffee consumption by regulating the expression *PDSS2* suggesting a possible functional explanation. The gene *PDSS2* codes for an enzyme responsible for the synthesis of the prenyl side chain of coenzyme Q10. Its mutation in mice leads to the insurgence of a renal phenotype (kd) which is characterized by tubulointerstitial nephritis, dilated tubules, and proteinuria^36^. In humans only one case of a *PDSS2* compound heterozygous mutant has been described in a child with Leigh syndrome, a very serious condition characterized by neonatal pneumonia, hypotonia, nephrotic syndrome and blindness^37^, although neither of the parents presented any symptoms at all. Further studies have shown that although the reduction in Q10 due to the *PDSS2* mutation is ubiquitous among the different tissues the observed phenotype is due to an augmented production of reactive oxygen species (ROS) specific to the affected tissues probably to the use of alternative mechanisms in the non-affected tissues^38^.

Conditional knockout of *PDSS2* in the liver has been shown to increase the expression of the genes of the caffeine metabolism pathway (GSEA normalized enrichment score 1.59, FDRp = 0.03) while on knock-out mice treated with probucol (which restores the knock-out phenotype) a strong inhibition was observed when compared to knockout mice (GSEA normalized enrichment score −1.87, FDR p = 0.02)^39^. We have shown through the comparison of our results with those of *PDSS2* eQTLs that in all examined tissues there is an inverse correlation between *PDSS2* expression and coffee consumption. Assuming that this is true also for the liver we may hypothesize that higher *PDSS2* expression would inhibit the expression of the genes in the caffeine metabolism pathway thus inhibiting caffeine degradation. The hypothesis of an eQTL in the liver is strengthened by the presence of one of our associated SNPs in an enhancer active in this specific tissue. This would fit very well with previous results that have associated speed in metabolizing caffeine and coffee intake through the *CYP1A2* genotype^18^,^19^. Unfortunately, liver eQTLs for *PDSS2* data on 1000G are not available yet and these results will need to be confirmed.

The difference in strength of effect between the Italian and the Dutch cohorts could be explained by differences in the way coffee is prepared in the two countries. In fact, while in Italy moka or espresso are the preferred way of drinking coffee, in the Netherlands filtered coffee is preferred. Although the concentrations of caffeine in the different preparation methods are similar, given the differences between cup sizes, Dutch intake of caffeine per cup is almost three times higher than Italians (average 173.25 mg/cup for filtered coffee vs. 67.18 and 60.95 for moka and espresso, respectively)^40^. It is thus possible that while *CYP1A2* genotype is important in determining coffee consumption at higher caffeine intakes, *PDSS2* may have a role at lower levels. According to this view we detected only an extremely mild association with previously reported *CYP1A2* SNPs in the Italian populations (rs2472297, *p* = 0.027). Of course further functional studies are needed to clarify this hypothesis.

Our results have highlighted a novel gene associated with coffee consumption adding new information towards understanding the genetic drivers of coffee consumption. The fact that this gene was not identified in previous much larger studies could be due to the use of a much denser SNP map (1000G) or to the fact that we have specified a different model of inheritance. Although further studies on larger cohorts will be needed to confirm our findings we believe to have added an important piece to the understanding of the genetic basis of coffee consumption and potentially to the mechanisms regulating caffeine metabolism.

## Additional Information

**How to cite this article**: Pirastu, N. *et al.* Non-additive genome-wide association scan reveals a new gene associated with habitual coffee consumption. *Sci. Rep.* **6**, 31590; doi: 10.1038/srep31590 (2016).

## Supplementary Material

Supplementary Information

Supplementary Dataset

## Figures and Tables

**Table 1:** Results from the association studies under the recessive genetic model.

SNP	Chr	Pos	A0	A1	β	SE	*p*	β rep	SE_rep	*p*_rep
6:107541346:I	6	107.5	S	L	0.086	0.014	1.05e-09	0.025	0.014	0.084
6:107546337:D	6	107.5	L	S	0.086	0.014	2.30e-09	0.024	0.015	0.112
rs12191399	6	107.5	T	C	0.086	0.014	9.84e-10	0.025	0.014	0.083
**rs2216084**	**6**	**107.6**	**C**	**T**	**0.082**	**0.014**	**9.25e-09**	**0.037**	**0.014**	**0.01**
rs4945774	6	107.5	C	A	0.086	0.014	1.04e-09	0.025	0.014	0.084
rs4946824	6	107.5	T	C	0.086	0.014	1.27e-09	0.026	0.015	0.085
rs6568476	6	107.5	T	C	0.084	0.014	2.75e-09	0.025	0.014	0.089
rs6568477	6	107.5	C	T	0.086	0.014	1.05e-09	0.025	0.014	0.085
rs6568479	6	107.5	A	G	0.086	0.014	8.91e-10	0.026	0.014	0.07
rs6915343	6	107.5	C	A	0.086	0.014	1.01e-09	0.024	0.014	0.086
rs6919118	6	107.5	T	C	0.086	0.014	1.06e-09	0.025	0.014	0.085
**rs6942255**	**6**	**107.6**	**G**	**A**	**0.082**	**0.014**	**9.79e-09**	**0.037**	**0.014**	**0.01**
rs7741724	6	107.5	G	A	0.086	0.014	1.05e-09	0.025	0.014	0.078
rs7741987	6	107.5	A	G	0.086	0.014	1.05e-09	0.025	0.014	0.085
**rs7745311**	**6**	**107.6**	**T**	**C**	**0.081**	**0.014**	**1.23e-08**	**0.037**	**0.014**	**0.008**
rs7749559	6	107.5	T	C	0.084	0.015	1.24e-08	0.03	0.015	0.055
rs7750154	6	107.5	T	G	0.086	0.014	1.14e-09	0.025	0.014	0.084
**rs7754744**	**6**	**107.6**	**A**	**G**	**0.082**	**0.014**	**8.80e-09**	**0.037**	**0.014**	**0.01**
rs9320207	6	107.5	T	C	0.086	0.014	1.11e-09	0.025	0.014	0.085
rs9372155	6	107.5	C	T	0.086	0.014	1.14e-09	0.025	0.014	0.085
rs9386627	6	107.5	T	C	0.086	0.014	1.01e-09	0.025	0.014	0.082
**rs9386630**	**6**	**107.6**	**T**	**G**	**0.082**	**0.014**	**1.07e-08**	**0.037**	**0.014**	**0.009**
rs9398123	6	107.5	A	C	0.086	0.014	1.13e-09	0.025	0.014	0.085
rs9398124	6	107.5	T	C	0.086	0.014	1.12e-09	0.025	0.014	0.085
rs9398125	6	107.5	A	C	0.086	0.014	1.05e-09	0.025	0.014	0.083
rs9398126	6	107.5	T	C	0.086	0.014	1.00e-09	0.025	0.014	0.082
rs9791276	6	107.5	C	T	0.086	0.014	1.15e-09	0.025	0.014	0.086
rs9791325	6	107.5	T	G	0.086	0.014	1.15e-09	0.025	0.014	0.086

Only SNPs significant at the discovery step were reported. Bold SNPs represent replicated SNPs. The β rep, SE rep and p rep columns represent the results for the replication analysis in the ERF cohort. Geneomic positions (Pos) are reported in Mb.
