# Supplementary material for: Non-additive genome-wide association scan reveals a new gene associated with habitual coffee consumption
Source: Sci Rep. 2016 Aug 25;6:31590. doi: 10.1038/srep31590 (PMC4997959; doi:10.1038/srep31590)
Supplement: Supplementary Information [file srep31590-s1.pdf]

Non-additive genome-wide association scan reveals a new gene associated with habitual coffee consumption

Nicola Pirastu[1,2,3*], Maarten Kooyman[4], Antonietta Robino[1], Ashley van der Spek[4], Luciano Navarini[7], Najaf Amin[4], Lennart C. Karssen[4,6], Cornelia Van Duijn [4,5], Paolo Gasparini[1,2].

1. Institute for Maternal and Child Health - IRCCS "Burlo Garofolo",2. University of Trieste, Italy, 3. Usher Institute of Population Health and Informatics, The University of Edinburgh, Edinburgh, UK., 4. Genetic Epidemiology Unit, Department of Epidemiology, Erasmus Medical Center, Rotterdam, the Netherlands, 5. Centre for Medical Systems Biology, Leiden University Medical Center, Leiden, The Netherlands, 6. PolyOmica, Groningen, The Netherlands, 7. illycaffe`s.p.a, Trieste, Italy.

Additional Tables.

Table S1. Cohort summary information

	N	Age mean(sd)	% Man	Coffee consumption cups/day mean (sd)
INGI-CARL	370	46 (16)	41.9	1.9 (1.6)
INGI-FVG	843	47.5 (14.8)	41.5	2.3(1.6)
ERF	1713	47.5 (12.8)	46.5	5.8 (3.6)

Table S2. Summary information for each GWAS performed in the discovery step.

	Model	N. SNPs	**λ**
INGI-FVG	addittive	6476444	1
	dominant	7049692	1.01
	recessive	3692733	1
INGI-CARL	addittive	9114185	0.99
	dominant	9022767	0.99
	recessive	5413630	1.02

Table S.3 Kendall Tau between the association pattern of coffee and the eQTL pattern in each of the Gtex database tissue.

Tissue	Kendall τ
Adipose Subcutaneous	-0.27
Artery Aorta	-0.44
Artery Tibial	-0.55
Cells Transformed fibroblasts	-0.38
Esophagus Mucosa	-0.61
Esophagus Muscularis	-0.52
Heart Left Ventricle	-0.44
Lung	-0.52
Muscle Skeletal	-0.52
Nerve Tibial	-0.49
Skin Sun Exposed Lower leg	-0.32
Stomach	-0.43
Thyroid	-0.39
Whole Blood	-0.32
